# Supplementary figures and images for: Built Environment Accessibility and Disability as Predictors of Well-Being among Older Adults: A Norwegian Cross-Sectional Study
Source: Int J Environ Res Public Health. 2023 May 20;20(10):5898. doi: 10.3390/ijerph20105898 (PMC10218673; doi:10.3390/ijerph20105898)

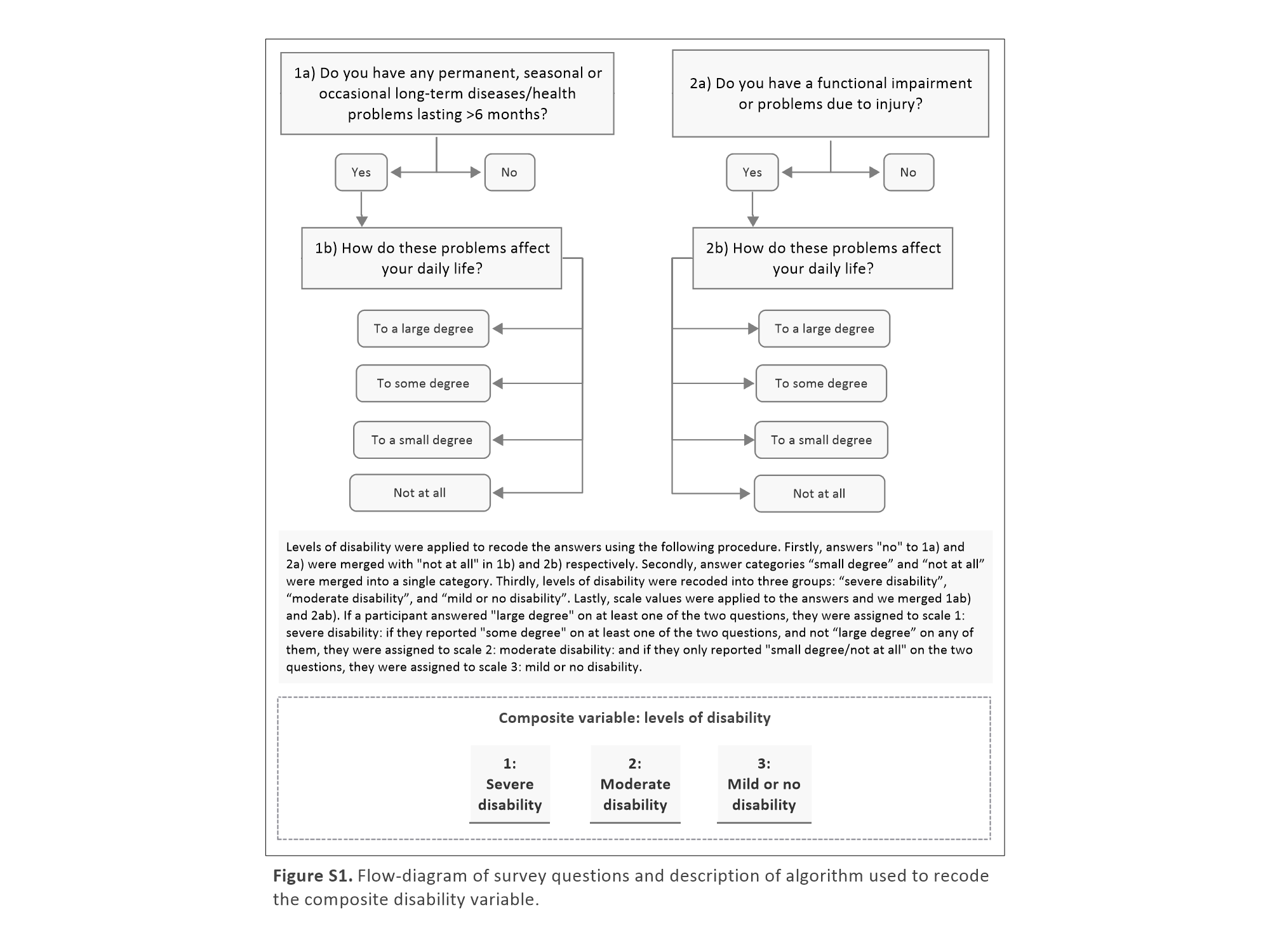

Supplement: Supplementary file 1 [file ijerph-20-05898-s001.zip › ijerph-2331856-supplementary.png]
